# Benefits and advances of Cone Beam CT use in prostatic artery embolization: review of the literature and pictorial essay

**DOI:** 10.1186/s42155-024-00459-1

**Published:** 2024-05-15

**Authors:** Nassir Rostambeigi, Daniel Crawford, Jafar Golzarian

**Affiliations:** 1 Vascular and Interventional Radiology, Mallinckrodt Institute of Radiology, Washington University, St. Louis, USA 510 South Kingshighway Blvd, MO 63110; 2 Vascular and Interventional Radiology, North Star Vascular & Interventional / University of Minnesota, Golden Valley, USA

**Keywords:** Prostatic artery embolization, Cone beam CT scan, Non-target embolization, Virtual injection

## Abstract

Prostatic artery embolization (PAE) has proven to be an efficacious treatment for urinary symptoms of benign prostatic hyperplasia. PAE is performed in a complex and challenging anatomical field which may pose difficulties from procedural standpoint. Cone beam computed tomography (CBCT) has been proposed as an invaluable tool during the PAE procedure. A review of different techniques and advancements, as well as demonstration of CBCT benefits via a pictorial overview of the salient examples is lacking. The techniques of CBCT are discussed herein and the virtual injection technology as an advancement in CBCT is discussed. To show the merits of CBCT in PAE, a pictorial overview of various clinical scenarios is presented where CBCT can be crucial in decision making. These scenarios are aimed at showing different benefits including identification of the origin of the prostatic artery and avoiding non-target embolization. Other benefits may include ensuring complete embolization of entire prostate gland as angiographic appearance alone can be inconclusive if it mimics a severely thickened bladder wall or ensuring adequate embolization of the median lobe to provide relief from “ball-valve” effect. Further examples include verification of embolization of the entire prostate when rare variants or multiple (> 2) arterial feeders are present.

## Evolution of the role of Cone Beam CT during PAE

Benign prostate hyperplasia (BPH) with lower urinary tract symptoms (LUTS) affects 80% of men over the age of 70 [1, 2]. Within the last two decades, prostatic artery embolization (PAE) has emerged as a minimally invasive method to treat moderate to severe LUTS [3–7]. Previous studies have demonstrated that prostate gland volume shrinkage, morphological changes, and tissue changes in the zones of prostate gland after PAE [8] translates into symptomatic improvement [9]. However, the recurrence of LUTS after PAE requiring re-intervention can be as high as 20% [10]. Several studies suggest that a complete coverage of the prostate gland may translate into more durable results. Unilateral embolization of the prostate gland has been frequently shown to be a risk factor for recurrence [11–13]. de Assis et al. suggested that lack of embolization of the posterolateral branch of prostatic artery could be a risk factor for repeat PAE [14]. The correlation between enlarged median lobe and bladder outlet obstruction also suggest that incomplete median lobe embolization can lead to incomplete clinical response [15, 16]. Additionally, Carnevale et al. demonstrated that proximal embolization of the prostatic artery followed by distal embolization reduces recurrence rates [17]. Finally, Hakime et al. showed a correlation between more contrast retention in the prostate gland and higher clinical success after PAE [18]. Technically successful PAE is therefore achieved by covering the entire prostate gland while avoiding non-target embolization.

Pelvic vasculature has a challenging anatomy due to arterial variations and extensive collateral flow [19, 20]. Cone beam computed tomography (CBCT) and post-processing image software have been shown to improve the identification of the prostatic artery origins [21, 22]. In addition, while increased operator experience the utilization of CBCT for identification of the prostatic arteries may become less essential [23], the importance of CBCT can still be seen in different scenarios. Avoiding non-target embolization is another important benefit of CBCT. Non-target embolization to the bladder, pudendal artery, or rectum can lead to adverse events such as bladder ulceration requiring surgical repair, penile ulceration, and rectorrhagia [11, 12, 22]. Prior studies have demonstrated some of the utilities from CBCT during PAE; in a study on 11 patients, CBCT was useful in identifying non-target embolization and to confirm adequacy of embolization [24]. However prior studies did not present a comprehensive review of the new techniques, along with a pictorial representation of different examples where CBCT was proven essential during PAE. The purpose of this review article is to provide an overview of the CBCT techniques described in the literature, present adjunctive technologies available for guidance, and provide illustrative examples where CBCT improved decision-making during PAE. Finally, the impact of CBCT on radiation dose during PAE is discussed and methods for radiation dose reduction are reviewed.

### CBCT Techniques

PAE procedure consists of a bilateral successive prostatic artery embolization. To delineate the anatomy, initial recommendations include digital subtraction angiograms (DSA) from the proximal internal iliac arteries at a 25–55-degree ipsilateral oblique projection with a 10 degrees craniocaudal angulation [25].

To facilitate and troubleshoot vascular anatomy in complex anatomical fields such as the pelvis, different imaging modalities and techniques have been described. Pre-procedural CT angiogram [26] and pre-procedural MR angiography (MRA) [27] with different benefits are described but are not within the scope of this review. Several other studies propose variations of the intra-procedural CBCT [21, 22, 24, 28–30] and additional virtual injection methods [20] in order to improve identification of prostatic arteries and treatment techniques (Tabe 1).

**Table 1:** Summary of different Cone Beam CT techniques

Contrast injection site	Injection rate	Injection volume	X ray delay	Advantages	Disadvantages
Aorta [23, 31, 39]	6–8 mL/S	50 mL (followed by 10 mL of saline) or 90 mL dilute contrast (60% contrast)	2–8 s	Able to demonstrate if PA origin is from external iliac artery	May obscure if there is cross coverage to the prostate lobes. May not show distal prostate perfusion well
Internal iliac artery[20, 22, 28, 29]	2–6 mL/S	22–26 mL	2–8 s	Able to identify multiple feeders	Non target embolization pattern can be different than prostatic artery injection. Expertise and software availability is required which could limit general use of virtual injection software
Super selective prostatic artery [21, 24]	0.5–1 mL/S	3–5 mL	4–10 s	Non-target pattern is similar to what will occur when particles are injected	Unable to detect multiple feeders to the prostate

*PA* Prostatic artery

CBCT techniques vary according to manufacturer and equipment and operator preferences. CBCT can be acquired with the arms down or with the arms on the patient’s chest with limited impact on image quality. CBCT can be obtained by injection from distal abdominal aorta [31], internal iliac arteries [32], and/or after selective catheterization of the suspected prostatic artery [32]. These variations are operator dependent and can have different values.

CBCT from distal aorta can be informative to show if the origin of the prostatic artery is from internal iliac or external iliac territory. However, this could fail to identify when there is overlap between right and left lobe perfusion which can only be detected if one side is injected. Distal perfusion to the prostate gland might be also suboptimal.

CBCT obtained from internal iliac artery combined with virtual injection software is valuable and can identify the multiple prostatic artery origins effectively. The downside could be that the actual site of the eventual particle injection site from prostatic artery is different than contrast injection site during CBCT, and this can change the non-target embolization pattern. Additionally, optimizing virtual injection software requires expertise and software availability to effectively assist in embolization process.

Finally, CBCT obtained from the prostate artery helps verify the presence of any non-target embolization from the actual treatment site and verifies whether there is complete coverage of the ipsilateral half of the prostate gland. Coverage of the median lobe can also be assured, and if the median lobe is not perfused by selective injection of the prostatic artery, then a search for a different feeder is warranted. The downside of CBCT obtained from prostatic artery is that previous DSA is needed to first identify the prostatic artery; if the prostatic artery is not correctly identified on CBCT, then more DSA images and CBCTs are needed to identify the correct artery, which will increase the dose and duration of the procedure.

Alternatively, a non-enhanced CBCT at the conclusion of the procedure can be performed which confirms adequate embolization; if there is incomplete embolization then further imaging is performed to identify other feeders and to assure complete embolization of the prostate.

Volume and rate of injection can vary between operators. At the author’s institution, 1 mL per second for 5 mL of full-strength contrast is injected through a 2.0 Fr microcatheter with the tip in the prostatic artery using a 4–6 s X-ray delay. The injection rate and volume may be modified to obtain optimum imaging in different situations. For example, larger volume with more X-ray delay can improve contrast uptake if the prostate size is markedly enlarged to allow time for contrast uptake by parenchyma. Alternatively, a slower injection rate may be considered for very small prostatic artery diameters.

Other groups reported different methods to acquire a CBCT using 3–5 mL of 50–70% concentration contrast and with hand- or power-injection at 0.2–1 mL per second with an 8–10 s X-ray delay [20]. The more delayed x-ray acquisition allows for improved perfusion of the prostate gland. A variation has been described with hand injection of 1.5–2 mL of full-strength contrast for 2–3 s with an imaging delay of 4–6 s [24]. A wall-mounted lead shield can be used in cases where the operator performs hand injection from within the room.

### CBCT and radiation dose considerations

The trade-off for using CBCT during PAE can be the increased radiation dose [23], but there are conflicting data between centers. Variations in CBCT technique to reduce radiation dose can play a role in different radiation doses reported. Additionally, centers with larger series reported that the use of CBCT can reduce the radiation dose by decreasing the number of DSAs needed during PAE [22, 33]. As discussed above each site of contrast injection for CBCT can have different values and the fact that larger centers reported less radiation may suggest that with more experience a smaller number of DSAs was required and overall dose was reduced. Thus, combination of smaller number of DSAs and judicious use of CBCT likely translate to lower overall radiation dose. Variations in technique such as CBCT performed using a 5-s rotation versus a 6-s rotation have shown similar diagnostic values but less radiation [34].

### Virtual injection technology

**Fig. 1:**
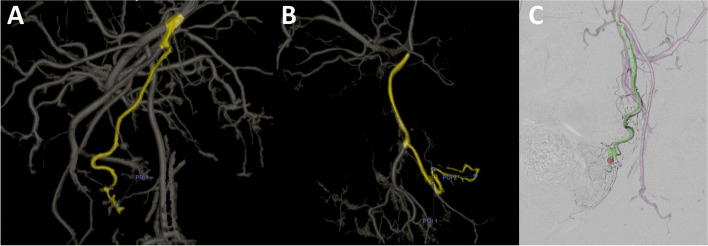
Virtual injection technology using GE Allia™ IGS 7 system. **A** 3D reconstruction after obtaining cone beam CT from internal iliac artery (2 mL/sec, total volume 24 mL, acquisition at 6 s delay), and then angulation was adjusted to best visualize the prostatic artery (POI = point of interest). **B** Virtual injection in another patient, with removal of the osseous structures to assist the visualization and identification of the prostatic artery. **C** 3D overlay can also be performed on live fluoroscopy to guide and facilitate the catheterization

In addition to the traditional CBCT usage during PAE, virtual injection software can automatically identify the vessels supplying the prostate gland, similar to the technology used for intra-arterial treatment of liver tumors. This can simulate embolization by using virtual injection points on a 3D reconstruction of vasculature from the CBCT data. Barrel et al. have shown that this technology can reduce the radiation dose by almost twofold [29] by reducing the number of DSAs needed. The software can also provide an overlay for the operator during fluoroscopy using the data from CBCT and provide a guide during cannulation (Fig. 1). The virtual injection software (Embo ASSIST® by GE Healthcare, or EmboGuide by Philips Healthcare) is also able to provide the best angle at which the prostatic artery can be visualized and cannulated, and this reduces the number of DSAs needed [28]. Another benefit of using virtual injection software is identification of the vessels that are sources of non-target embolization. As discussed above, a possible downside regarding the use of virtual injection software is the necessary staff and software availability and that subtle motion artifact or suboptimal phase of the contrast injection can change the final CBCT quality and decision making.

### Illustrative examples demonstrating different scenarios with CBCT utility

**Fig. 2:**
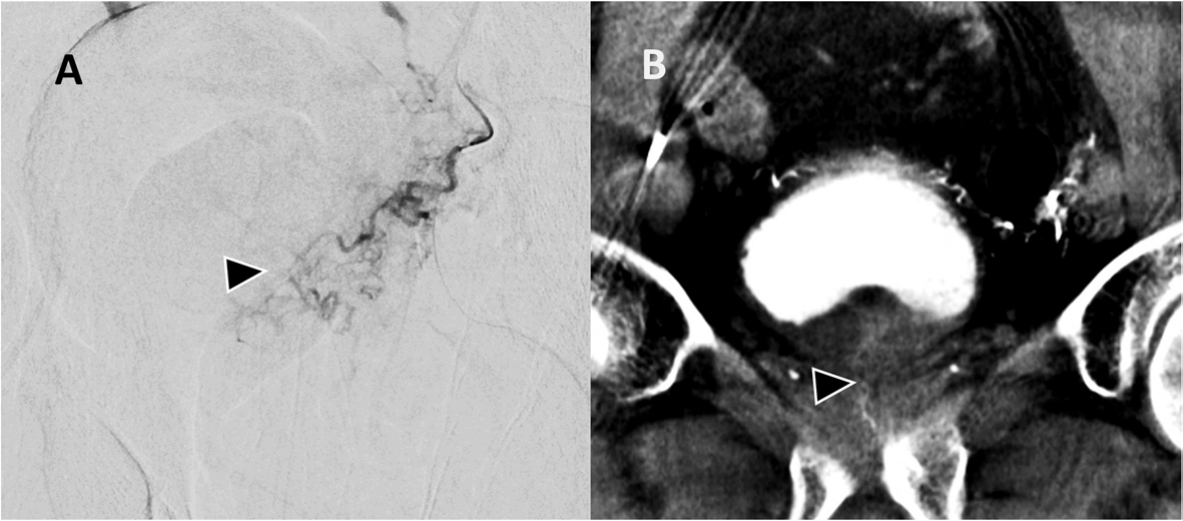
**A** Selective DSA of the left prostatic artery (black arrowhead) with a typical course of prostatic artery but the perfusion blush is not convincingly depicting perfusion of the prostate. This was not a very enlarged gland (70 mL). B) CBCT confirmed perfusion of the left lobe of the prostate (black arrowhead). Small vesical branches seen at the dome of the bladder are due to reflux from power injection during CBCT and were not visualized or included during embolization

**Fig. 3:**
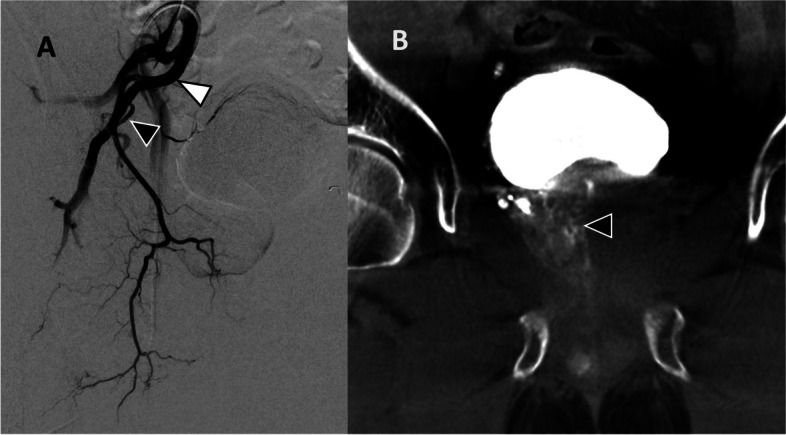
**A** DSA of the right internal iliac artery (white arrowhead) showed a variant right prostatic artery arising from the right inferior gluteal artery (black arrowhead). **B** CBCT after cannulation of the variant right prostate artery confirmed perfusion of the right lobe (black arrowhead). Prostate was not extremely enlarged (73 mL) and the prostate blush could be inconclusive without CBCT confirmation

**Fig. 4:**
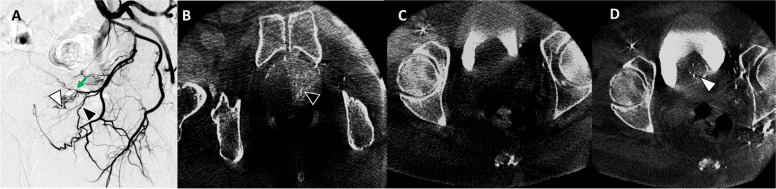
**A** Left internal iliac artery angiogram demonstrated prostatic artery (black arrowhead) arising off the left obturator artery. **B** After the successful left side embolization, then the right side was embolized. **C** CBCT without contrast injection at completion was attempted to ensure adequate coverage. This did not show coverage for the median lobe. **D** Then further review showed an accessory prostatic artery from the left superior vesical artery (green arrow). The branch of superior vesical artery (white arrowhead in panel A) then showed perfusion of the median lobe which was confirmed on CBCT (white arrowhead)

**Fig. 5:**
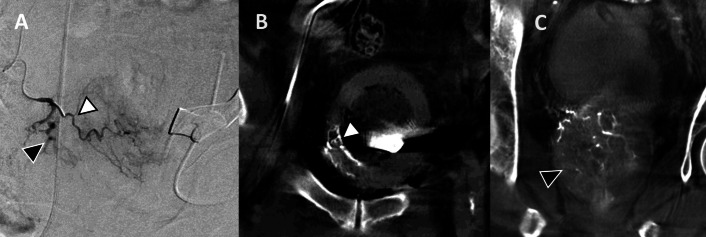
**A** Initial DSA of right internal iliac artery demonstrated a typical prostatic artery origin from the superior vesical artery and a possible prostate blush (open arrowhead). **B** However, CBCT demonstrates that this blush was the thickened bladder wall (white arrowhead). **C** The more proximal branch (black arrowhead in panel A) of this same artery was in fact perfusing the prostate (black arrowhead)

**Fig. 6:**
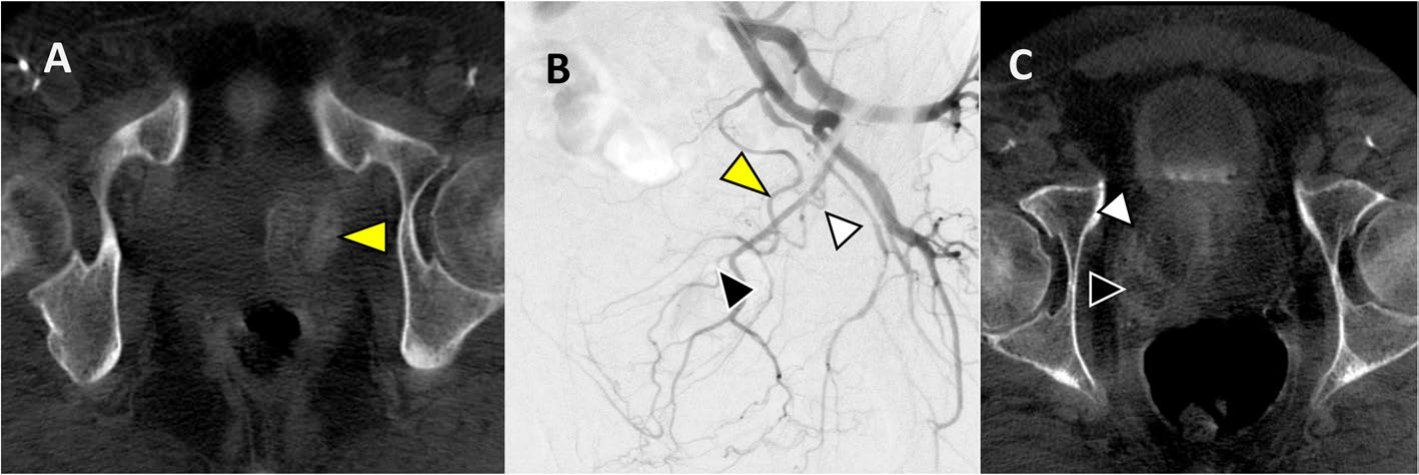
**A** Left prostatic artery was originating from superior vesical artery and was successfully embolized (yellow arrowheads in A and B). However, the main right prostatic artery, which was also originating from superior vesical artery (not shown), did not cover the right lobe entirely. **B** Further scrutiny showed a branch of left internal pudendal artery (white arrowhead) and left obturator artery (black arrowhead) that were perfusing the right lobe (**C**)

**Fig. 7:**
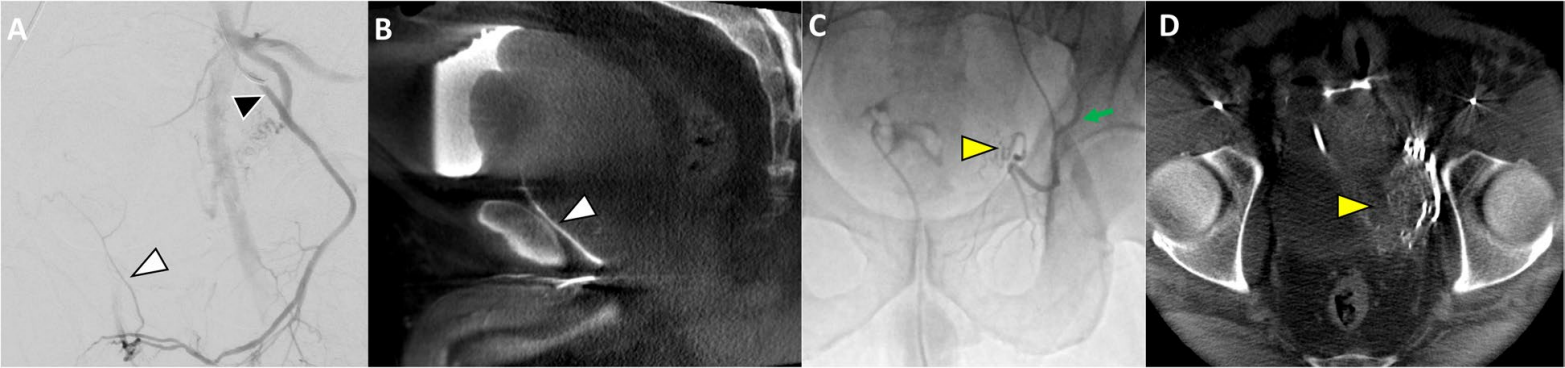
**A** Selective DSA of the left internal pudendal artery (black arrowhead) did not reveal a definite prostatic artery or obturator artery. A terminal branch of the left internal pudendal artery is seen extending cephalad (white arrowhead). **B** Sagittal reconstruction of a contrast enhanced CBCT with selective injection in this terminal branch (white arrowhead) demonstrated perfusion of the median lobe. C&D) evaluation of the left inferior epigastric artery was then performed (green arrow), which confirmed that the prostatic artery (yellow arrowhead) was arising from the aberrant left obturator artery (corona mortis). Interestingly, this patient also had a right prostate artery origin from an aberrant right obturator artery (not shown)

**Fig. 8:**
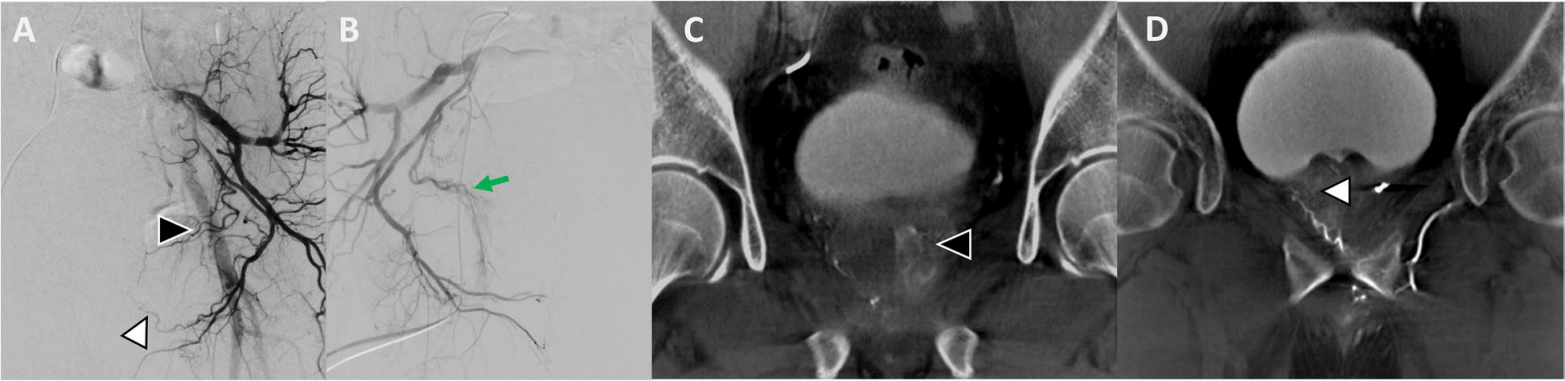
**A** Selective DSA of the left internal iliac artery demonstrates the left prostatic artery (black arrowhead) arising off the internal pudendal artery. **B**&**C** The right prostatic artery was explored from the right internal iliac artery. A possible prostatic artery (green arrow) was interrogated and was found to perfuse the rectum rather than the prostate. Note the staining of the left lobe of the prostate from the successful embolization of left prostate artery. **D** Selective CBCT from the terminal branch of the left internal pudendal artery (white arrowhead in panel A) shows perfusion of the right prostate hemisphere

**Fig. 9:**
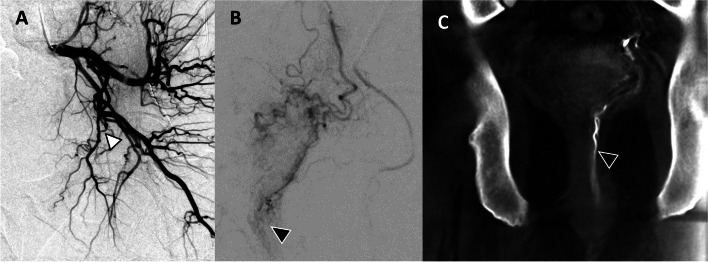
Left prostatic artery identified arising off the left internal pudendal artery (white arrowhead). **B** Selective DSA from the left prostatic artery shows a possible rectal supply from the capsular branch of the prostatic artery (black arrowhead). **C** This is confirmed on CBCT (black arrowhead). Subsequently the rectal branch was coiled before safe embolization of the prostate

Multiple scenarios are presented to illustrate how CBCT can improve decision making during PAE. Emphasis is placed on ensuring adequate prostate gland coverage while avoiding non-target embolization, focusing on cases where DSA alone can be likely inconclusive. These examples are not the only scenarios where CBCT can be essential in decision-making. The examples include ensuring complete treatment of the entire prostate including the median lobe and moderate-sized prostate gland Figs. 2, 3, 4 and 5), identifying arterial anatomic variants and multiple feeding arteries (Figs. 6, 7,8), and avoiding non-target embolization to the bladder (Fig. 5), rectum (Fig. 9) or penis. Bladder non-target embolization can sometimes result in bladder wall necrosis requiring surgical intervention [35] and on DSA a severely thickened bladder can mimic a contrast blush of moderately enlarged prostate gland.

### Complete prostate gland coverage

Moderate-sized prostate glands (< 80 mL) can be challenging to identify on DSA and difficult to differentiate from the adjacent bladder wall. CBCT allows confirmation that the catheterized vessel supplies the prostate gland rather than adjacent structures. Figure 2 demonstrates a moderate-sized prostate with typical course of the prostatic artery, but the prostate blush was difficult to ascertain on DSA alone. If adequate prostatic coverage had not been confirmed with CBCT then further searching would have been performed. Similarly, Fig. 3 demonstrates a case where there is a rare variant anatomy in addition to a prostate gland volume of less than 80 mL making the prostate blush less conclusive and hence a CBCT was helpful for effective embolization. As seen later in Fig. 5, a thick bladder wall can mimic the prostate blush and could be inadvertently embolized, and CBCT can distinguish between the two.

### Median lobe coverage

Enlargement of the median lobe of the prostate can lead to intravesical prostatic protrusion and result in LUTS secondary to bladder outlet obstruction with a “ball-valve” phenomenon [36]. Ensuring adequate embolic coverage of the median lobe is therefore essential to adequately treat patients with intravesical prostatic protrusion and sufficiently relieve bladder outlet obstruction.

The median lobe coverage can be difficult to verify on DSA alone, and there can be incomplete median lobe coverage. One method that has been described is to perform CBCT without contrast injection which can confirm adequate median lobe coverage at the conclusion of the procedure. As seen in Fig. 4, CBCT performed at the conclusion of embolization helped identifying incomplete coverage of the median lobe and prompted an additional search for accessory prostatic arteries from the left vesical artery. As shown in this example, CBCT changed the decision making by demonstrating lack of median lobe coverage from the initial treatment and later confirmed sufficient median lobe coverage.

### Anatomic variants

**Fig. 10:**
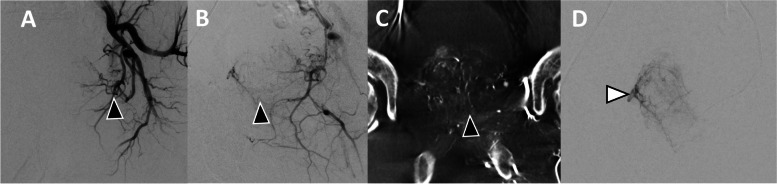
**A** Left internal iliac artery DSA shows left prostatic artery origin from obturator artery (black arrowhead). **B** After selective DSA of left prostatic artery it is suspected that both lobes are being supplied by this artery (black arrowhead). **C** CBCT confirmed perfusion of bilateral lobes of the prostate (black arrowhead). **D** Subsequently selective contralateral right-side embolization was performed (white arrowhead) followed by embolization of the left prostate gland

A wide variety of anatomical variants for prostatic blood supply have been described in the literature [37]. In patients with variant anatomy, identifying the arteries that supply the prostate can be perplexing, and CBCT allows confirmation for a safe and effective treatment. Examples of variants may include prostatic supply arising from branches of the external iliac artery (Fig. 7), origin of the prostatic artery from posterior division branches (Fig. 3), bilateral supply of both prostate hemispheres from one internal pudendal artery (Fig. 8) or obturator artery (Fig. 10), or prostatic artery origin from femoral artery [38]. CBCT helps ensuring the complete perfusion of the prostate gland and identify sources of non-target embolization in these scenarios.

### Multiple feeding arteries to prostate

It is not uncommon to have multiple arteries supplying the prostate gland, especially in patients with atherosclerotic disease leading to stenosis and/or occlusion of the typical prostatic arteries. This can be difficult to discern with DSA alone. By performing CBCT, incomplete coverage of the prostate can be identified, which would prompt the search for additional arteries supplying the prostate; subsequently, CBCT can convincingly show complete coverage of the entire prostate gland (Fig. 6).

### Avoiding non-target embolization

The bladder is one of the most frequent sites of non-target embolization during PAE [24]. Enhancement of a thickened bladder wall can easily mimic the prostate gland blush, especially in a moderate-sized prostate gland where distinguishing between prostate gland and thickened bladder wall could be challenging (Fig. 5). CBCT allows confirmation of prostate perfusion and identification of potential non-target bladder wall perfusion. Additionally, the prostate itself sometimes has a unique shape and orientation, which can simulate the bladder wall as seen in Fig. 5; in this case, embolization was not performed without CBCT confirmation. Of note, other common non-target sites include the rectum (Fig. 9) or penis, which have classic angiographic patterns of a vertically orienting artery towards the rectum or antero-medially towards the penis respectively. In these scenarios, DSA can be more conclusive.

## Conclusion

While DSA can be sometimes sufficient to identify the prostatic arteries, the use of CBCT can considerably assist in PAE by providing additional information that is not obtained from DSA alone and reduce procedural time and radiation and increase efficiency. The scenarios presented are several examples where CBCT improved decision making. In summary, CBCT and virtual injection technology can ensure prostate is completely treated including coverage for median lobe, and additionally provide information for patients who have an anatomic variant or have multiple feeding arteries to the prostate, while identifying the sources of non-target embolization.

## Data Availability

All materials for this manuscript are available upon request.
